# Empowering patients: how accurate and readable are large language models in renal cancer education

**DOI:** 10.3389/fonc.2024.1457516

**Published:** 2024-09-26

**Authors:** Abdulghafour Halawani, Sultan G. Almehmadi, Bandar A. Alhubaishy, Ziyad A. Alnefaie, Mudhar N. Hasan

**Affiliations:** ^1^ Department of Urology, King Abdulaziz University, Jeddah, Saudi Arabia; ^2^ Mohammed Bin Rashid University of Medicine and Health Sciences, Dubai, United Arab Emirates; ^3^ Department of Urology, Mediclinic City Hospital, Dubai, United Arab Emirates

**Keywords:** artificial intelligence, kidney cancer, patient education materials, health literacy, large language models, accuracy, readability

## Abstract

**Background:**

The incorporation of Artificial Intelligence (AI) into healthcare sector has fundamentally transformed patient care paradigms, particularly through the creation of patient education materials (PEMs) tailored to individual needs. This Study aims to assess the precision and readability AI-generated information on kidney cancer using ChatGPT 4.0, Gemini AI, and Perplexity AI., comparing these outputs to PEMs provided by the American Urological Association (AUA) and the European Association of Urology (EAU). The objective is to guide physicians in directing patients to accurate and understandable resources.

**Methods:**

PEMs published by AUA and EAU were collected and categorized. kidney cancer-related queries, identified via Google Trends (GT), were input into CahtGPT-4.0, Gemini AI, and Perplexity AI. Four independent reviewers assessed the AI outputs for accuracy grounded on five distinct categories, employing a 5-point Likert scale. A readability evaluation was conducted utilizing established formulas, including Gunning Fog Index (GFI), Simple Measure of Gobbledygook (SMOG), and Flesch-Kincaid Grade Formula (FKGL). AI chatbots were then tasked with simplifying their outputs to achieve a sixth-grade reading level.

**Results:**

The PEM published by the AUA was the most readable with a mean readability score of 9.84 ± 1.2, in contrast to EAU (11.88 ± 1.11), ChatGPT-4.0 (11.03 ± 1.76), Perplexity AI (12.66 ± 1.83), and Gemini AI (10.83 ± 2.31). The Chatbots demonstrated the capability to simplify text lower grade levels upon request, with ChatGPT-4.0 achieving a readability grade level ranging from 5.76 to 9.19, Perplexity AI from 7.33 to 8.45, Gemini AI from 6.43 to 8.43. While official PEMS were considered accurate, the LLMs generated outputs exhibited an overall high level of accuracy with minor detail omission and some information inaccuracies. Information related to kidney cancer treatment was found to be the least accurate among the evaluated categories.

**Conclusion:**

Although the PEM published by AUA being the most readable, both authoritative PEMs and Large Language Models (LLMs) generated outputs exceeded the recommended readability threshold for general population. AI Chatbots can simplify their outputs when explicitly instructed. However, notwithstanding their accuracy, LLMs-generated outputs are susceptible to detail omission and inaccuracies. The variability in AI performance necessitates cautious use as an adjunctive tool in patient education.

## 1 Introduction

Renal cell carcinoma (RCC) constitutes roughly 2% of all cancer diagnoses and mortalities worldwide (1). Over the past five decades, its incidence has doubled in developed nations (2). There has been a noticeable increase in the incidental finding of renal tumors owing to the widespread use of thoracoabdominal imaging for unrelated issues (3). As a result, most incidental kidney cancers are small, localized, and asymptomatic (4). The management options for renal cell carcinoma are developing rapidly with the new interest in the molecular characterizations of RCC subtypes which lead to potential new therapeutic options. Subsequently, patients may encounter significant decisional conflict when confronted with a plethora of management options.

In the context of kidney cancer, patient education is of a paramount importance in elucidating the disease states and the corresponding management strategies. Various forms of patient education, including face-to-face sessions, video or audio recordings, electronic materials, and printed information materials, are available. Given the constraints one-on-one clinical time, patients and physicians commonly depend on printed kidney cancer patient education materials (PEMs) to support clinical decision-making and to elevate patient’s comprehension of the entire range of treatment options. Despite the authoritative nature of these PEMs issued by prominent urological organizations, their comprehensibility by patients remains a question. Patients may also seek to augment their understanding of available treatment options through self-directed internet searches.

With the current emergence of artificial intelligence (AI) and large language models (LLM) as a source of information, a revolutionary impact is noticeable in all aspects of our daily life, and medical field is not an exception. However, the accuracy and comprehensibility of these new forms, the “chatbot,” has not been fully addressed in this space. Examples of some popular LLM systems include ChatGPT which is the most widely used LLM, it is constructed upon the Generative Pre-trained Transformer (GPT) linguistic processing models, aims to produce output that closely mimic human language (5). Gemini AI, formerly known as Google Bard, has the same aims as ChatGPT, but it belongs to LaMDA family of large language models (6). Perplexity AI, designed to handle extensive volume of information, are trained using large-scale datasets sourced from the internet and leverage advanced natural language processing (NLP) techniques to answer queries effectively (7).

Our study aimed to assess the readability and accuracy of patient-related content on kidney cancer provided by publicly accessible LLMs. We conducted a comprehensive comparison with the PEMs provided by internationally recognized organizations, including the American Urological Association (AUA) and the European Association of Urology (EAU). Additionally, we explored the potential of LLMs to simplify output for improved readability. The results of our research will shed light on the quality of information and misinformation disseminated by AI chatbots. This will provide a valuable guide for physicians, directing patients to reliable and easily understandable sources of information related on kidney cancer.

## 2 Materials and methods

This study was determined to be exempt from the Institutional Review Board approval as it did not involve the use of any patient data. The study was designed based on previous research that looked into the accuracy and readability of content produced by publicly accessible chatbots and websites (8–12).

Patient education materials (PEMs) from the American Urological Association (AUA) and the European Association of Urology (EAU) pertaining kidney cancer were evaluated and sorted into four topics: general information, renal cancer diagnosis, treatment, and follow-up. Google Trend was employed to ascertain the most prevalent search queries related to kidney cancer. From this analysis, the top five search results for “Patient Questions Kidney Cancer” were reviewed, and the nine most commonly recurring questions were selected for evaluation (Appendix I).

The study involved inputting each of the nine questions directly into ChatGPT- 4.0, Perplexity AI, and Gemini AI, replicating the way patients may use chatbots to seek medical advice. These questions were sorted into one of the four predefined categories. The same prompts were entered into the chatbots, instructing them to provide responses at a sixth-grade literacy level. In this study, we utilized a Likert scale to evaluate the accuracy of chatbot-generated responses, offering more flexibility than traditional binary approaches. This method captures varying degrees of agreement and highlights areas of consensus or disagreement, allowing for a comprehensive assessment of the material’s reliability. Four independent urologists assessed the responses using a 5-point scale, focusing on five key aspects: 1) comprehensiveness in describing the subject, 2) adherence to established guidelines, 3) existence of inaccuracies, 4) relevance and applicability of the outputs, and 5) inclusiveness across diverse populations, in alignment with previous studies (10, 12). The responses were then scored on a scale from 1 to 5: 1 for accurate and complete, 2 for accurate but omitting minor detail, 3 for mostly accurate or omitting multiple minor details, 4 for mostly inaccurate or omitting significant detail, and 5 for inaccurate. Each reviewer rated the nine questions on a five-point scale according to the previously established scoring methodology. Following this, the average score for each AI chatbot’s was determined, and an overall average, combining all reviewers’ scores, was calculated to obtain the median score for each LLM. The PEMs from AUA and EAU were deemed to have an accuracy score of 1.

To assess the readability of the LLMs responses and official PEMs, we utilized verified readability formulas including Gunning Fog Index (GFI), Simple Measure of Gobbledygook (SMOG), and Flesch-Kincaid Grade Formula (FKGL). These indices are widely used and validated tools for evaluating English texts, specifically designed to determine the US literacy proficiency level of inputted texts. They consider various factors, such as word and sentence length, syllable per word, and the proportion of simple to complex words. An online calculator, available at (https://www.webfx.com), was utilized to calculate scores of inputted texts. Each readability score correlates with a particular grade reading level, Lower scores indicate simpler texts that are easier to read, while higher scores suggest more complex texts requiring advanced reading skills. For instance, a score of 6 indicate that a sixth-grade education level is needed. The average of these scores was computed to determine the mean reading level, corresponding to a particular literacy proficiency level

Statistical analysis: Data were coded, tabulated and analyzed using (SPSS) version 26. Quantitative data was expressed as mean and standard deviation (Mean ± SD).

## 3 Results

The results of our analysis revealed distinct performance characteristics among the three artificial intelligence systems evaluated: ChatGPT-4.0, Perplexity AI, and Gemini AI.

In terms of readability, evaluated based on SMOG, GFI, and FKGL scores, a wide range of variability has been noticed across all categories. Gemini AI presented the most readable content across a range of categories including General Information and Diagnosis. Specifically, in the General Information category, Gemini AI achieved a readability score of 9.15 ± 2.04, which was lower than ChatGPT-4.0’s score of 9.84 ± 1.96 and Perplexity AI’s score of 12.62 ± 7.65, Similarly, in the diagnosis category, both Gemini AI (9.85 ± 2.32) and ChatGPT-4.0 (10.37 ± 2.06) outperformed Perplexity AI (12.02 ± 2.97) in terms of readability (**Table 1**). However, the Follow-up outputs showed the highest readability scores compared to all other categories, where ChatGPT-4.0 scored 12.61 ± 3.04, Perplexity AI scored 15.74 ± 9.58, and Gemini AI achieved a score of 13.94 ± 1.57. Despite Gemini AI’s generally has lower readability scores, ChatGPT-4.0 produced the most readable content in both the Treatment and Follow-Up categories, 8.80 ± 2.72 and 12.61 ± 3.04 respectively (**Table 1**).

**Table 1:** Mean accuracy and readability scores of ChatGPT- 4.0, Perplexity AI and Gemini AI generated responses to kidney cancer prompts.

Input topic	ChatGPT- 4.0						Perplexity AI							Gemini AI					
	SMOG	GFI	FKGL	Mean ± SD	Accuracy	Mean Accuracy	SMOG	GFI	FKGL	Mean ± SD	Accuracy	Mean Accuracy	SMOG		GFI	FKGL	Mean ± SD	Accuracy	Mean Accuracy
**Geneal information**	11.7	10.02	7.8	9.84 ± 1.96	1.4	1.5	14.94	16.53	10.3	12.62 ± 7.65	1.7	2.2	11.34		8.8	7.3	9.15 ± 2.04	1.6	1.9
**Diagnosis**	12.5	10.2	8.4	10.37 ± 2.06	1.6		14.42	12.93	8.7	12.02 ± 2.97	1.7		12.24		9.7	7.6	9.85 ± 2.32	2.2	
**Treatment**	13.34	13.68	8.8	8.80 ± 2.72	2		13.02	11.79	10.1	11.64 ± 1.47	3.1		14.61		12.25	9.4	12.09 ± 2.61	2.0	
**Follow-up**	14.42	14.3	9.1	12.61 ± 3.04	1.2		18.77	22.08	12.7	15.74 ± 9.58	2.2		15.25		14.38	12.2	13.94 ± 1.57	1.7	

(Accuracy 1-5, 1= accurate and complete, 2= accurate but omitting minor detail, 3= mostly accurate or omitting multiple minor details, 4= mostly inaccurate and/or omitting significant detail, 5= inaccurate) SMOG, Simple Measure of Gobbledygook; GFI, Gunning Fog Index; FKGL, Flesch Kincaid Grade Level.

Moreover, ChatGPT-4.0 showcased the highest accuracy across all the categories, with a mean of 1.**5** (**Table 1**). For instance, in the diagnosis category, ChatGPT-4.0 obtained an accuracy score of 1.6, indicating higher accuracy compared to Perplexity AI at 1.7 and Gemini AI at 2.2 (**Table 1**). In the Treatment category, Perplexity AI achieved an accuracy score of 3.1, while both ChatGPT-4.0 and Gemini AI scored 2.0. In the General Information category, ChatGPT-4.0 scored 1.4, Perplexity AI with 1.7 and Gemini AI with 1.6.

As an additional analysis, AI Chatbots were instructed to simplify their responses to target a Grade 6 reading level. Overall, LLMs successfully reduced the readability scores; however, a grade 6 reading level was not achieved uniformly across all categories. For General Information, ChatGPT-4.0 achieved a mean score of 5.76 ± 1.49, which was lower than both Perplexity AI’s 8.27 ± 1.89 and Gemini AI’s 6.43 ± 1.56. In the Diagnosis category, ChatGPT-4.0 scored 5.94 ± 1.34, Perplexity AI at 8.45 ± 1.55 and Gemini AI at 6.61 ± 1.51 (**Table 2**).

**Table 2:** Mean readability scores of generated responses by ChatGPT-4.0, Perplexity AI, and Gemini AI at a grade 6 reading level for various topics related to kidney cancer, assessed using the Simple Measure of Gobbledygook (SMOG), Gunning Fog Index (GFI), and Flesch Kincaid Grade Level (FKGL).

Input topic	ChatGPT – 4.0				Perplexity AI				Gemini AI			
	SMOG	GFI	FKGL	Mean ± SD	SMOG	GFI	FKGL	Mean ± SD	SMOG	GFI	FKGL	Mean ± SD
**Geneal information**	7.40	5.39	4.50	5.76 ± 1.49	9.10	9.60	6.10	8.27 ± 1.89	8.02	6.38	4.90	6.43 ± 1.56
**Diagnosis**	7.42	4.80	5.60	5.94 ± 1.34	10.24	7.50	7.60	8.45 ± 1.55	8.21	6.42	5.20	6.61 ± 1.51
**Treatment**	10.23	8.44	8.90	9.19 ± 0.93	8.05	8.30	7.05	7.80 ± 0.66	10.04	8.66	6.60	8.43 ± 1.73
**Follow-up**	9.83	7.20	7.20	8.08 ± 1.52	7.60	7.80	6.60	7.33 ± 0.64	6.00	7.10	8.30	7.13 ± 1.15

Furthermore, the Treatment category showing that Perplexity AI had the best readability mean score of 7.80 ± 0.66, which was lower than ChatGPT-4.0 at 9.19 ± 0.93 and Gemini at 8.43 ± 1.73. Moreover, In the Follow-Up category, the Gemini AI’s readability score was 7.13 ± 1.15, compared to Perplexity AI at 7.33 ± 0.64 and ChatGPT-4.0 at 8.08 ± 1.52.

**Figure 1:**
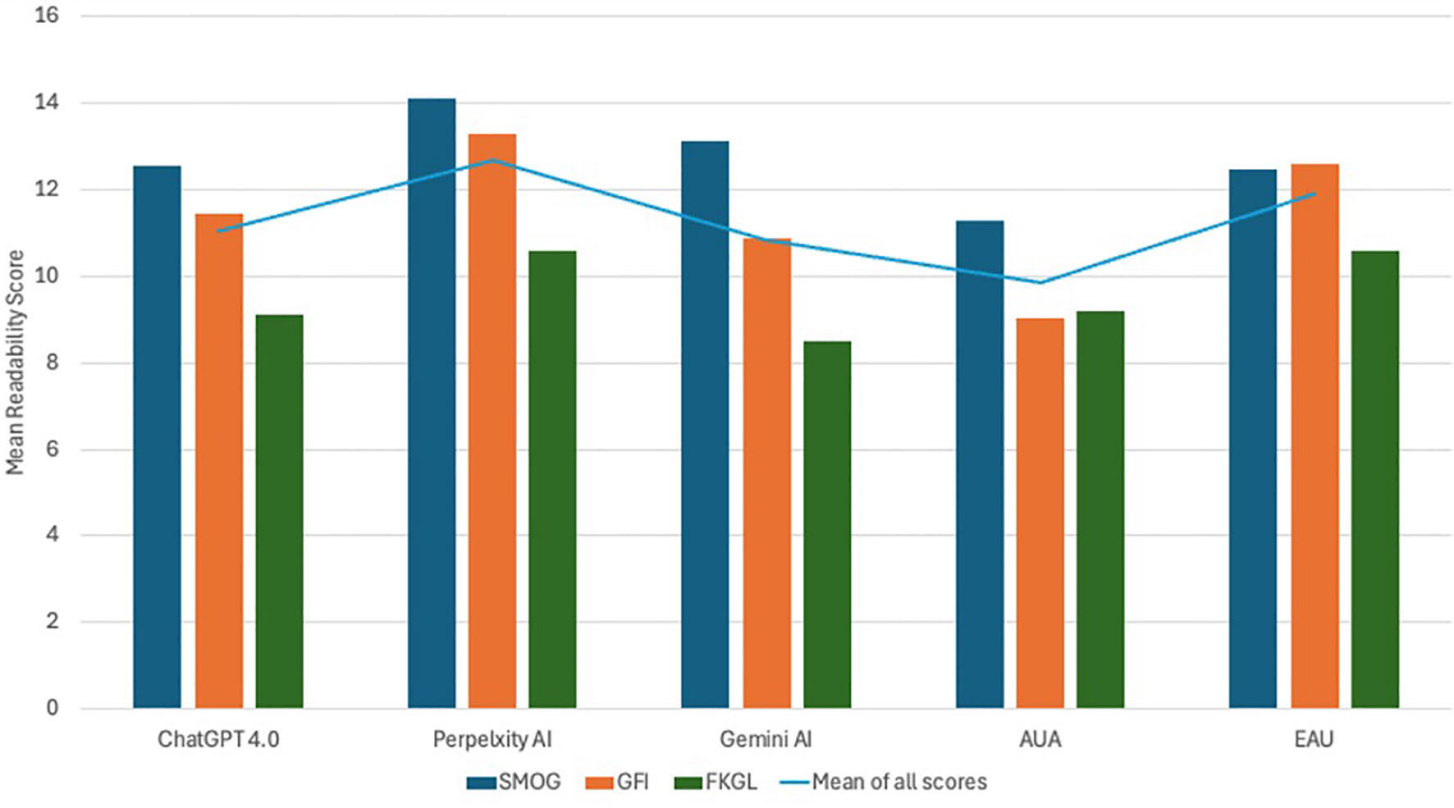
Illustrates the mean readability scores of patient educational materials (PEMs) sourced from the American Urological Association (AUA) and the European Association of Urology (EAU) in comparison to the responses generated by prominent large language models - ChatGPT-4.0, Perplexity AI, and Gemini AI.

When comparing AI-generated contents against patient education materials (PEMs) from recognized urological bodies, including the American Urological Association (AUA) and the European Association of Urology (EAU), we observed that the AI contents generally exhibited higher readability scores, suggesting higher complexity. Specifically, PEM published by AUA was the most readable, with a mean readability score of 9.84 ± 1.2 compared to ChatGPT-4.0 (11.03 ± 1.76), and EAU’s (11.88 ± 1.11). Perplexity AI demonstrated the highest complexity with a mean readability score of 12.66 ± 1.83, whereas Gemini AI had a mean score of 10.83 ± 2.31 (**Figure 1**).

## 4 Discussion

The role of artificial intelligence continues to expand in healthcare practice. LLMs have garnered significant attention as a valuable resource for patients seeking to acquire medical knowledge about their health conditions. Generally, Caregivers rely on PEMs published by official health organizations as an accurate and up-to-date adjunct to ensure patients’ comprehension of discussed information during one-on-one clinical time. Unfortunately, patients recall barely a fifth of provided health information, while 40-80% of the discussed content will be immediately forgotten (13). Hence, printed materials can significantly aid in patients’ memorization of health information and subsequently better adherence to the treatment (14). However, the complexity of written information may represent a real challenge, especially for vulnerable patients, those with low education and literacy, non-native speakers, refugee, elderly, and those with low socio-economic status (15, 16). A nationwide study in the U.S. revealed an intermediate level of health literacy among the majority of adult population (53%), while 36% had bare or below basic levels (17). It is crucial to understand these limitations to ensure that patients can engage adequately and make informed decisions. On that account, it is strongly advised that written patient education material be composed at a sixth-grade reading level to ensure comprehensibility (18, 19).

The official patient education materials (PEMs) are essential, but healthcare professionals should also consider other sources of health information. The capacity to search for and obtain health information online has become increasingly vital in contemporary healthcare. Both caregivers and patients are increasingly utilizing the internet as a source for health-related information. In recent years, online health information seeking behavior has become a global trend (20, 21). In Europe, 55% of the adult population sought medical-related information online in a 2020 survey, showing a significant 21% increase since 2010 (20). In the U.S. the percentage of online health information seekers reached 74.4% in 2017, up from 61.2% in 2008 (22). Seeking health information online is believed to have profoundly positive impact on patients’ healthcare decisions, treatment adherence, and management choices (23–25). However, a considerable variation in the quality and readability of medical online information has been reported (10, 11, 26–28). Numerous studies have indicated that online information regarding orthopedic injuries, benign prostatic hyperplasia, and pancreatic cancer has a poor readability (10, 11, 29). Notable disparity in the quality of medical online content is evident as well. Bouhadana et al. confirmed a high accuracy of online information materials related to BPH (10), while Storino et al. demonstrated a lack of accurate knowledge regarding pancreatic cancer therapy (11).

Large language models (LLMs) are sophisticated AI systems engineered to analyze extensive datasets to generate a human-like language. These LLMs, such as ChatGPT, Gemini AI, and Perplexity AI, are becoming increasingly popular due to their interactive nature, allowing users to ask questions and receive detailed and tailored responses (5–7). Given the popularity of these new technologies, studies have already begun to evaluate their efficacy in responding to medical queries. Within the field of urology, A recent study found that ChatGPT provided highly accurate (71.1– 94.3%) prostate cancer-related information; however, the technology failed to deliver patient-friendly content (30). Musheyev et al. evaluated AI chatbot responses to urological cancer queries, finding that while accurate, the information was difficult to read and lacked clear user instructions (8). Additionally, ChatGPT delivered highly precise responses to thirty questions covering a range of urological conditions and pediatric urology (31, 32).

Considering the increasing use of these technologies, our study aimed to evaluate the accuracy and readability of various LLMs in providing kidney cancer-related information, expanding beyond a singular focus on ChatGPT.

The results of our assessment of the accuracy of Chatbots in providing information about kidney cancer, using a 5-point Likert scale, revealed a generally high level of output accuracy. However, some minor details were found to be omitted. Among the Chatbots, ChatGPT 4.0 demonstrated slightly better performance compared to Perplexity AI and Gemini AI, with accuracy scores of 1.5, 2.2, and 1.9, respectively. It’s important to note that while Chatbots may excel in certain tasks, their accuracy in other areas may be lower (30, 33). The underlying reasons for this performance disparity remain unclear; however, it may stem from inherent limitations in their ability to grasp context, subtleties, and the complexities of human language. Tasks that demand profound comprehension, empathy, or innovative problem-solving pose significant challenges for chatbots, as they depend on pre-established algorithms and training data that may not fully encapsulate the nuances of every scenario. Moreover, chatbots often encounter difficulties with ambiguous queries, dynamic language evolution, or situations that fall outside their training scope, resulting in less precise or pertinent responses (34). For instance, we found that the responses related to kidney cancer treatment and follow-up provided by the Chatbots were less accurate, as they were often difficult to understand and ambiguous. Nevertheless, LLMs are not devoted to Information inaccuracy; Gemini AI, for example, provided answers that did not fully adhere to official guidelines, such as stating “A biopsy is the only definitive way to diagnose kidney cancer.” This could potentially lead to increased frustration for patients upon diagnosis. Misinformation generated by LLMs, referred to as “ Artificial Hallucinations,” occurs when false or misleading citations are outputted, further complicating their application in a medical context (34). Unlike ChatGPT, illustrative images were included in the outputs of both Gemini AI and Perplexity AI in response to various queries. The influence of these visual and multimedia elements necessitates further assessment by patients to fully ascertain their additional impact on patient comprehension.

In order to enhance patient comprehension of written education materials, it is imperative to adhere to the advised 6^th^-grade reading level, thereby ensuring that the content is accessible and easily understandable to broader audience. Our analysis revealed overall higher than recommended readability scores for the responses generated by LLMs (ChatGPT 11.03 ± 1.76, Perplexity 12.66 ± 1.83, and Gemini AI 10.83 ± 2.31). Of the official urological organizations, PEMs created by AUA have the lowest readability scores (9.48 ± 1.27) compared to EAU (11.88 ± 1.11) and AI chatbots responses. Moreover, a variable performance was noticed when LLMs were asked to simplify the text to a sixth-grade level. Our study showed that while LLMs generally did well at making text more readable for the general public, some categories still remained above the recommended literacy level. In a study by Moons et al. ChatGPT was able to simplify patient information materials, but it did not achieve the 6^th^ – grade reading levels, while Google Bard (Gemini AI) oversimplified the texts by omitting 83% of the content (35). On the other hand, ChatGPT successfully reduced the complexity of orthopedic surgery PEMs to the sixth-grade level (36). There is no clear explanation for the variation of the in ChatGPT’s capability to simplify some texts but not others. Overall, more work is needed to ensure that patient education materials meet the recommended readability level.

This study is subject to several limitations. Firstly, there is no universally accepted and well-recognized instrument to assess the quality of outputs generated by LLMs. The inherent variability of this AI technology could potentially result in different outputs depending on the input provided. Therefore, the results of this study are limited to the prompts utilized here as input. Secondly, as this technology advances, conducting the same study using an updated version of AI chatbots may lead to different results as indicated by other studies (12, 37). Third, the readability formulas do not account for the potential impact of figures and diagrams on overall patient comprehension, which may give certain AI an advantage over others. Finally, incorporating patient’s evaluations of various PEMs alongside our assessment tools would provide more accurate pragmatic insights. Therefore, additional research is recommended to elucidate the distinctions between these perspectives.

## 5 Conclusion

The current study provided valuable insights into the readability and quality of kidney cancer information produces by various LLMs. Among all the materials evaluated, the PEM published by the AUA is the most readable. Nevertheless, it is crucial to acknowledge that the readability levels of all examined texts remain elevated above the recommended literacy threshold for the general population. While AI Chatbots exhibits commendable capacity to simplify complex texts, they encounter challenges in uniformly achieving the 6^th^-grade level in some categories. Despite the high accuracy levels characterizing the outputs generated by LLMs, they are not entirely immune to detail omission and informational inaccuracies. This inherits variability in performance calls for cautious use of LLMs as supplementary tools alongside conventional ones.

## Data Availability

The original contributions presented in the study are included in the article/supplementary material. Further inquiries can be directed to the corresponding author.

## Appendix

The most searched questions pertaining kidney cancer and their categories

**Table:**

Searched questions	Input topic	Categories
Q1	What is the function of the kidneys?	General information
Q2	What are kidney masses?	General information
Q3	What causes kidney masses?	General information
Q4	What are the symptoms of kidney cancer?	General information
Q5	Diagnosis of kidney cancer	Diagnosis
Q6	What does kidney tumor grade and stage mean?	Diagnosis
Q7	What is the treatment of kidney cancer?	Treatment
Q8	Follow-up of localized kidney cancer	Follow-up
Q9	Treatment of metastatic kidney cancer	Treatment
